# New Jersey Center for Tourette Syndrome Sharing Repository: methods and sample description

**DOI:** 10.1186/1755-8794-1-58

**Published:** 2008-11-26

**Authors:** Gary A Heiman, Robert A King, Jay A Tischfield

**Affiliations:** 1 Department of Genetics, Rutgers University, 145 Bevier Road, Piscataway, NJ 08854, USA; 2 Child Study Center of Yale University, New Haven, CT, USA

## Abstract

**Background:**

Tourette Syndrome is a neuropsychiatric disorder characterized by chronic motor and phonic tics. Affected individuals and their family members are at an increased risk for other neuropsychiatric conditions including obsessive-compulsive disorder and attention deficit hyperactivity disorder. While there is consistent evidence that genetic factors play a significant etiologic role, no replicable susceptibility alleles have thus far been identified.

**Description:**

Here we discuss a sharing resource of clinical and genetic data, the New Jersey Center for Tourette Syndrome Sharing Repository, whose goal is to provide clinical data, DNA, and lymphoblastoid cell lines to qualified researchers.

**Conclusion:**

Opening access to the data and patient material to the widest possible research community will hasten the identification of causal genetic factors and facilitate better understanding and treatment of this often impairing disorder.

## Background

Tourette Syndrome (TS) is a neurological disorder characterized by chronic motor and phonic tics that make their appearance before 18 years of age [1]. Prevalence ranges from 1–3% and the disorder is found across many ethnic groups around the world [2]. Males are affected 3 to 4 times more often than females, and the condition is less often diagnosed in adults than in children, most likely because tics often spontaneously diminish or disappear by later adolescence [3]. Individuals with TS and their family members frequently exhibit comorbid difficulties such as obsessions and compulsions (including full-blown OCD), which may reflect alternative manifestations of the genetic factors; ADHD; uneven neuropsychological profile (e.g., visual-motor integration and fine-motor skill deficits); and vulnerability to anxiety and depression are also common [4-8].

Family, segregation and twin studies consistently indicate that genetic factors play a significant role in the etiology of TS [for reviews, see [9-11]]. Although there have been some initial positive findings [12-15], identification of replicable susceptibility alleles has thus far remained elusive. Possible explanations for non-replication include phenotypic and locus heterogeneity, gene-environmental interaction, population stratification, and differences in phenotype definition or model specification [9,11,16-19]. There is a need for large replication samples. Open exchange of clinical data and biomaterials can foster new and innovative research that may overcome these putative impediments and may encourage more investigators to work on Tourette Syndrome [20-22]. To facilitate the clinical data and DNA samples from well-characterized Tourette Syndrome probands and their families reaching the widest possible research community, we established a sharing resource of clinical and genetic data, the New Jersey Center for Tourette Syndrome Sharing Repository (NJCTS Sharing Repository). The purpose of the repository is to identify genetic factors that play a causal role in Tourette Syndrome (TS), Chronic Motor or Vocal Tic Disorder, and associated disorders such as Chronic Tics (CT), Obsessive-Compulsive disorder (OCD) and Attention Deficit Hyperactivity Disorder (ADHD) by providing clinical data, DNA, and/or lymphoblastoid cell lines to qualified researchers from throughout the world. This article describes repository's data collection procedures and clinical assessments as well as how researchers can access the data.

## Construction and content

### Subjects and methods

Subjects were recruited from the Tourette Syndrome Association of New Jersey (TSANJ) membership and from clinicians in New Jersey, New York, and Connecticut. The study was approved by the Rutgers University institutional review board; all subjects or their legal guardians gave informed written consent (and for minors, written assent) to participate. Potential subjects (or their legal guardian) were recruited by telephone. Subjects were informed that the repository is open to qualified researchers from around the world and that no personally identifiable information (as defined by Health Insurance Portability and Accountability Act) or link to identifying information is included in repository. Consenting subjects (or their legal guardian) completed a set of self-report questionnaires, were evaluated at the Rutgers University Tourette Syndrome Clinic by a child psychiatrist experienced in the diagnosis of TS and related disorders, and a DNA sample was obtained most frequently from blood and rarely from saliva. Probands were included if they met strict DSM-IV-TR criteria [1] for TS or Chronic Motor or Vocal Tic Disorder. Probands not meeting this inclusion criterion are excluded from the repository. Family members (first and second degree) were also recruited if they reported having had any tics or OCD. In addition, we attempted to recruit first-degree relatives of the proband regardless of any tic or psychiatric symptomatology.

### Assessments

#### Self-report

The self-report questionnaire [23] includes sub-scales assessing tic disorders, OCD, and ADHD. The tic disorder sub-scale was adapted from Yale Global Tic Severity Scale (YGTSS) [24]. For OCD, we used the Dimensional Yale-Brown Obsessive-Compulsive scale [25]. The adult self-report questionnaire included an ADHD symptom checklist, whereas the parent-on-child questionnaire includes a version of the SNAP-IV ADHD rating scale [26], with additional items concerning age of onset, impairment, setting, and history of prior diagnosis or treatment. The self-report also includes sections assessing medical history, relevant medication use, birth and development, and family history. The adult and parent-on-child self-report questionnaires are available on the NJCTS repository website at: .

#### Clinical diagnosis

Final diagnoses were made according to strict DSM-IV-TR criteria [1] by an experienced child psychiatrist (RK) specializing in Tourette Syndrome and were based upon review of the self-report forms followed by direct semi-structured interview of the subject or parent informant. Each subject was assessed for the presence or absence of the following conditions: TS or other tic disorders (including chronic motor or vocal tic disorder, transient tic disorder, and tic disorder NOS), OCD or obsessive-compulsive symptomatology (including subclinical OCD, obsessive-compulsive symptoms, or obsessive-compulsive personality disorder), ADHD (including combined, inattentive, hyperactivity/impulsivity subtypes), and trichotillomania. For each condition for which symptoms were reported, the level of diagnostic certainty was rated as ("Definite", "Probable", or "Possible") based on the completeness and unambiguity of the symptom reports, agreement between informants, and extent to which diagnostic criteria were met [27,28]. The diagnostician also rated tic and OCD severity using the YGTSS Tic Severity Rating scale [24] and DYBOCS Score Sheet [25]. The Summary Rating Form, the YGTSS Tic Severity Rating scale, and DY-BOCS Score Sheet are available for download from NJCTS repository website.

To identify atypical TS cases or cases which occurred in conjunction with potentially confounding conditions, the cases were routinely "flagged" for the following: atypical presentation (e.g. tics not appearing until late in adolescence or after head trauma), psychosis, pervasive developmental or a Autism spectrum disorder, other neurological problems, congenital anomalies, or suspected genetic syndrome/chromosomal abnormalities. Since these potentially confounding conditions (e.g., Austim spectrum) were not the primary diagnoses of interest, no specialized instruments were used to make these diagnoses. If, in the course of the developmental/medical history, the subject or parent reported other current or lifetime diagnoses, these were briefly queried, noted, and flagged. Diagnoses and flags were coded on the Summary Rating Form and entered into a database.

#### Creation of permanent cell lines

Blood specimens collected from participating subjects are brought to the Rutgers University Cell Repository at Rutgers University (RUCDR), where EBV-transformed lymphoblastic permanent cell lines are created, frozen, thawed, and re-grown to ensure viability. DNA is extracted from these cell lines for genetic studies.

#### Sample description

Study recruitment began in June, 2007 and is ongoing. Our current goal is to collect DNA, cell lines and clinical information from approximately 1000 persons who either seem to have TS or are related to someone with TS. In addition, we are currently assembling a research team whose goal is to submit a multi-site collaboration grant to substantially increase the sample size. For a summary of the current data collection, see the NJCTS repository website at: . As of November 1^st^, 2008, a total of 75 (23 female and 52 male) probands with TS or Chronic Motor or Vocal Tic Disorder have been evaluated and provided DNA. Of these, 10 are singletons (i.e., no other relatives included) and 65 have at least one other relative included (i.e., denoted as pedigree families). The 65 pedigree families comprise of 47 trios (proband plus both biological parents) for association analyses. Of the 65 pedigree families, 253 subjects were evaluated and 245 provided DNA. Of these, 86 subjects met criteria for definite TS and 96 met criteria for TS or other tic disorders at the probable or definite certainty level.

## Utility and discussion

The NJCTS Sharing Repository contains clinical data, DNA, and lymphoblastoid cell lines on singleton probands, trios (probands plus both parents), and larger pedigrees. All subjects, both affected and unaffected, are evaluated by an experienced psychiatrist specializing in Tourette Syndrome. Data collection is ongoing and submitted samples are available for distribution as soon as they have been processed and the accompanying clinical/demographic data entered (i.e., no embargo period). For a summary of the current data collection, clinical assessment forms, instructions for accessing the data, see the NJCTS repository website at: .

## Conclusion

The NJCTS Sharing Repository provides a unique resource for disseminating clinical data, DNA, and lymphoblastoid cell lines to qualified researchers interested in conducting genetic analyses on Tourette Syndrome and associated conditions. The goal is to provide data access to the widest possible research community to hasten the identification of causal genetic factors and facilitate better understanding and treatment of this often impairing disorder.

## Availability and requirements

NJCTS Sharing Repository is devoted to the collection and expedited distribution of biomaterials and clinical data for the genetic analysis of Tourette Syndrome and related disorders. Therefore, there is no embargo period between sample submission and availability for distribution. Anonymous data on family structure, age, sex, clinical status, and diagnosis ("clinical data"), DNA samples, blood plasma, and cell line cultures ("biomaterials") are distributed to qualified researchers at recognized biomedical research facilities. Researchers may gain access to clinical data, genetic analysis data, and biomaterials after receiving approval based on the qualifications of the investigators in conducting genetic research on complex disorders. Once approval is given, clinical data and biomaterials are sent after payment of a modest access fee. As of 4/1/08, we have distributed clinical data and DNA to three independent international research groups. For more instructions on how to obtain access to data, see the NJCTS repository website at: .

## Authors' contributions

GAH participated in the design and coordination of the repository and drafted the manuscript. RAK participated in the design, carried out the clinical assessments and helped draft the manuscript. JAT conceived, participated in obtaining funding, and in design of the repository. JAT also participated in critical revisions of all of the submitted publication material. All authors read and approved the final manuscript.

## Pre-publication history

The pre-publication history for this paper can be accessed here:


